# HCG Trigger of GnRH Agonist-Induced Functional Ovarian Cysts Does Not Decrease Clinical Pregnancy Rate in GnRHa Pretreated Frozen Cycles: Evidence From a Retrospective Cohort Study

**DOI:** 10.3389/fendo.2022.876517

**Published:** 2022-06-15

**Authors:** Hong Zeng, Chen Zhang, Lei Zhang, Nenghui Liu

**Affiliations:** ^1^ Department of Reproductive Medicine Center, Xiangya Hospital, Central South University, Changsha, China; ^2^ Department of Reproductive Medicine Center, Changsha Maternal and Child Health Care Hospital, Hunan Normal University, Changsha, China

**Keywords:** gonadotrophin releasing hormone agonist (GnRH agonist), functional ovarian cyst, frozen-thawed embryo transfer, hormone replacement therapy (HRT), propensity score matching

## Abstract

**Background:**

GnRH agonist (GnRHa) pretreatment before the frozen-thawed embryo transfer (FET) was increasingly utilized. However, the incidence of GnRHa-induced functional ovarian cysts (FC) was inevitable. The feasibility and efficacy of HCG triggering GnRHa-induced FC are unknown.

**Objective:**

The aim of the study was to investigate the effect of HCG triggering GnRHa-induced FC on FET outcomes.

**Methods:**

A total of 657 HRT-FET cycles with GnRHa pretreatment were retrospectively analyzed. Patients were divided into the FC group and the no functional cysts (NC) group according to whether the patient developed FC (follicular diameter of ≥7 mm and E_2_ of ≥100 pg/ml). Risk factors associated with the incidence of GnRHa-induced FC were determined by multivariate regression analysis. Pregnancy outcomes were compared between the FC group and the NC group. Propensity score matching (PSM) was performed to reduce the impact of confounding factors. Three multivariate regression models were performed to assess the association between HCG triggering GnRHa-induced FC and clinical pregnancy. Interactive analysis and subgroup analysis were also analyzed.

**Results:**

The incidence rate of GnRHa-induced FC was 9.74%. Older age (aOR 1.10, 95% CI 1.05-1.15, p-value < 0.001) and lower BMI (aOR 0.81, 95% CI 0.71-0.93, p-value=0.002) are risk factors for GnRHa-induced FC. The implantation rate, clinical pregnancy rate (CPR), and miscarriage rate were not significantly different between the FC group and the NC group before or after PSM (p-value > 0.05). Multivariate logistic models showed that HCG triggering GnRHa-induced FC does not decrease CPR in the general population (p-value > 0.05). The effect of HCG triggering GnRHa-induced FC on clinical pregnancy is interactive with age (p-value for interaction: 0.003); HCG trigger is associated with significantly higher CPR than HRT-FET cycles without FC in patients ≥35 years (aOR 4.40, 95% CI 1.57–12.3, p-value = 0.005).

**Conclusions:**

HCG triggering GnRHa-induced FC does not decrease the chance of clinical pregnancy in HRT-FET cycles pretreated with GnRHa.

## Introduction

The selection of an appropriate endometrial preparation strategy is crucial for frozen embryo transfer (FET). There are four commonly used endometrial preparation strategies, namely, the natural cycle (NC)-FET, ovulation stimulation-FET, hormone replacement therapy (HRT)-FET, and HRT with GnRHa pretreatment (GnHRa + HRT-FET). No evidence supports significant differences in pregnancy rates between the above FET strategies (1). Clinically, the FET strategy was selected based on the complications of the patient (endometriosis, PCOS, or recurrent implantation failure) and the experience of the clinician. The NC-FET applies to patients with regular ovulation. Compared to NC-FET, the ovulation stimulation regime can be used for patients with abnormal ovulation while increasing the risk of OHSS due to multiple follicle development (2). A simple HRT regime has the advantages of flexible and shorter endometrial preparation time and lower cost (3). However, it cannot avoid unexpected luteinizing hormone (LH) rises, which may disturb the implantation window and affect pregnancy outcomes. GnRHa pretreatment is widely used for pituitary downregulation in FET. It is associated with a higher implantation rate and clinical pregnancy rate, partly due to improved endometrial receptivity (4). Though the effect of GnRHa pretreatment before FET is conflicting (5–13), some studies have shown an increased tendency of clinical pregnancy rate following GnRHa pretreatment before FET cycles in patients with endometriosis, PCOS, or RIF (9–13). However, the formation of functional ovarian cysts (FC) is one of the common complications following pituitary down-regulation using GnRHa.

The incidence rate of FC ranges from 6.42 to 39% (14–17). The different incidence rates may be related to the different timing of B-ultrasound monitoring after downregulation, different dosage, or administering strategy of GnRHa, and the pretreatment with oral contraceptives (14, 18). FC can be induced following different protocols (follicular-phase GnRHa protocol or luteal-phase GnRHa protocol) and different GnRH agonist types (short-acting GnRHa or long-acting GnRHa). The causes of FC, on the other hand, are unknown. Possible explanations include the transient flare-up effect of GnRHa on gonadotrophins, insufficient suppression of the circulating gonadotrophins to hypophysectomy levels, the direct effect of GnRHa on the ovaries and steroidogenesis, and the serum progesterone level at the time of GnRHa administration (16, 19–21).

Some previous studies support that FC does not impact IVF outcomes (20, 22, 23). Other studies suggest that FC decreases the quality and number of oocytes retrieved, the fertilization rate, the number and quality of embryos, implantation rates, and pregnancy rates (16, 24). Cyst aspiration does not improve IVF outcomes (16, 25). The FC may negatively affect IVF outcomes by mechanically reducing the space for growing follicles, impairing the ovarian blood supply, or decreasing oocyte quality. However, in FET cycles, we do not acquire oocytes; whether there is a harmful effect of the FC on FET outcomes is unknown. Moreover, evidence has shown that the FC may contain mature follicles, and successful pregnancy can be achieved by HCG triggering oocyte maturation in fresh cycles (26–28). Whether the HCG-triggering GnRHa-induced FC is applicable in FET cycles remains an open question. Therefore, investigating the effect of GnRHa-induced FC on FET outcomes is urgently needed. This study aims to investigate the risk factors associated with the incidence of GnRHa-induced FC and the effect of HCG triggering GnRHa-induced FC on FET outcomes.

## Materials and Methods

### Ethics Approval

The Ethics Committee of Xiangya Hospital approved the study (accession number 2021035). Since this is a retrospective analysis of data routinely collected from treatments and patients, informed consent was waived by the Ethics Committee of Xiangya Hospital.

### Patients and Study Design

**Figure 1:**
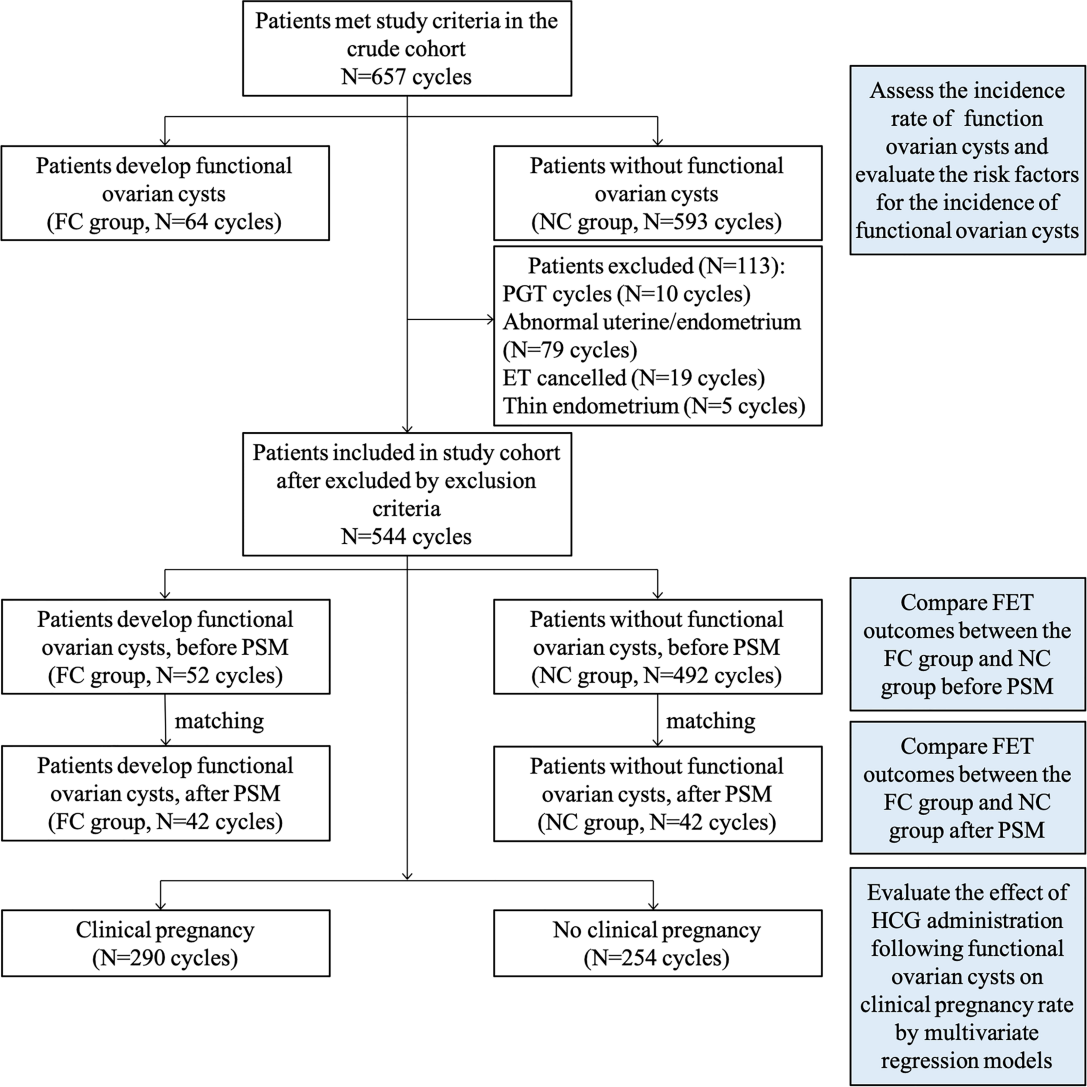
The flowchart of the study.

The study was a retrospective cohort study consisting of 599 patients who underwent 657 HRT-FET cycles pretreated with 3.75 mg GnRHa from 2018 to 2020. The inclusion criteria are: (1) patients planned for HRT-FET cycles with 3.75 mg GnRH agonist pretreatment in the early follicular phase; (2) the timing of GnRHa downregulation is from days 2 to 5 of menstruation; and (3) the luteal support protocol is the standard protocol in our center. The exclusion criteria are (1) PGT cycles or oocyte donor cycles; (2) patients with intrauterine adhesion, uterine myoma, untreated endometrial polyps, endometritis, uterine malformations; (3) exogenous estrogen is given to promote endometrial growth in patients who develop functional ovarian cysts; (4) primary variables are missing; and (5) embryo transfer canceled due to no transferable embryo, uterine effusion, or thin endometrium (<7 mm). After excluding patients within the exclusion criteria, 544 cycles remained for further statistical analysis. The patients were divided into the functional cysts (FC) and the no functional cysts (NC) groups according to whether the patient developed FC following GnRHa pretreatment. The functional ovarian cyst was defined as the following: a thin-walled intra-ovarian sonolucent structure with a mean diameter of ≥17 mm and serum estradiol (E_2_) levels ≥100 pg/ml. No functional ovarian cysts were defined as patients without ovarian cysts or patients with ovarian cysts but whose plasma estradiol (E_2_) level was not elevated (<100 pg/ml). A diagram of this study is shown in **Figure 1**.

### FET Protocol and Endometrial Preparation

**Figure 2:**
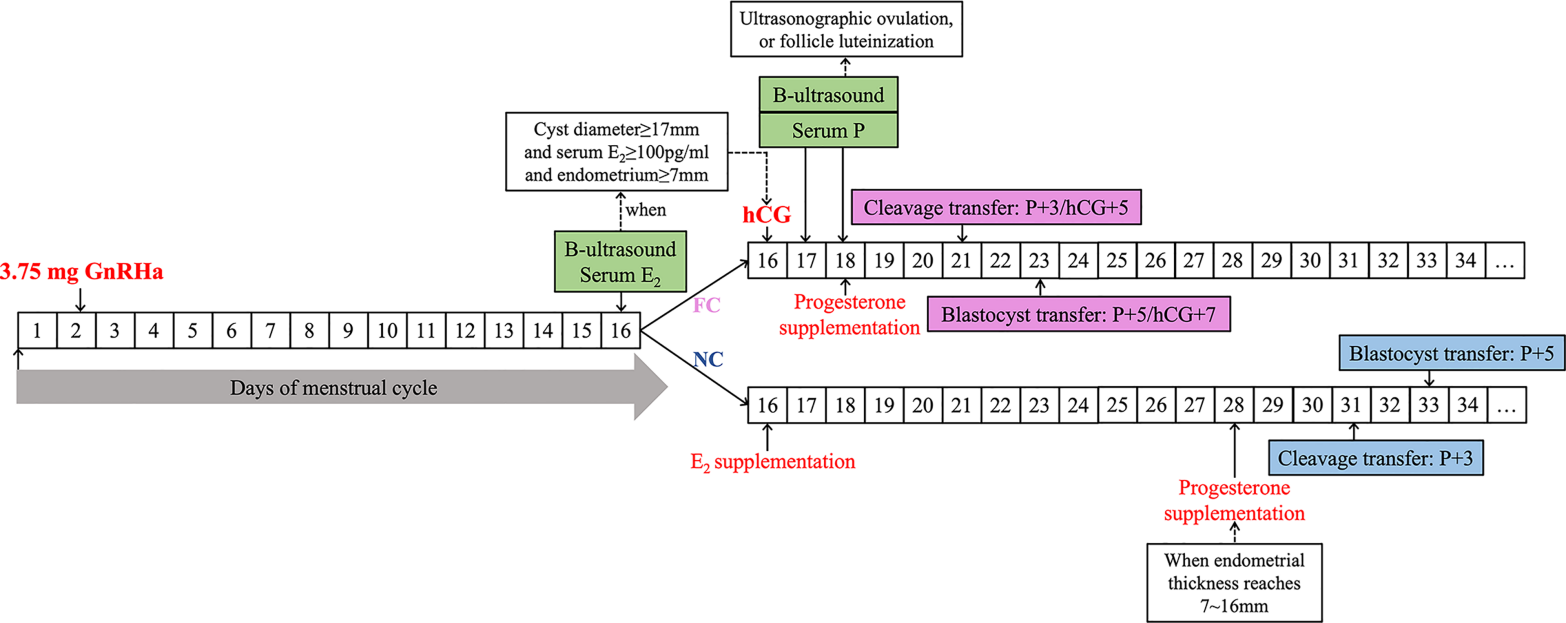
The flowchart of HRT-FET cycles pretreated with 3.75 mg GnRHa.

Approximately 3.75 mg of GnRHa was given on day 2 (a range from days 2 to 5 is applicable) of the menstrual cycle. Ultrasound was performed 14 days after GnRHa downregulation (GnRHa + 14) to screen FC. If there is a follicle cyst, ultrasound monitoring is performed every day. An injection of 10,000 IU of human chorionic gonadotropin (HCG, Lizhu Corp., China) was used to induced ovulation until the follicle diameter ≥17 mm and E_2_ level ≥ 100 pg/ml. The patient underwent ultrasound and serum progesterone (P) detection the day after HCG injection (HCG + 1) and two days after HCG injection (HCG + 2). Progesterone supplementation (luteal phase support) starts immediately (HCG + 2) when we observe ultrasonographic ovulation or follicular luteinization with serum P4 level increases. Then the cleavage-stage embryo was transferred three days after progesterone supplementation (P + 3, or HCG + 5), and the blastocyst was transferred five days after progesterone supplementation (P + 5, or HCG + 7). No exogenous estrogen was supplemented in patients with FC. For patients without FC, exogenous estrogen was supplemented 14 days after GnRHa downregulation (GnRHa + 14) to promote endometrial growth. The patient underwent ultrasound screening one week after E_2_ supplementation to check the endometrial thickness; the E_2_ dose was adjusted based on endometrial thickness. Ultrasound screening was performed every two to three days afterwards. Progesterone was given to transform the endometrium when the endometrial thickness reaches 7 to 16 mm and the duration of exogenous E_2_ exposure is lasted for at least 12 days. Then the cleavage embryo was transferred on P+3, and blastocyst was transferred on P+5. The flowchart of the protocol is shown in **Figure 2**. In the flowchart, the timing of the GnRHa injection on Day 2 was taken as an example.

### Luteal Phase Support

The luteal phase was supported by 600 mg progesterone capsules orally per day (200 mg per time, three times a day) combined with 200 mg progesterone capsules vaginally per day.

### Outcomes and Definitions

The primary outcome is the clinical pregnancy rate (CPR). The secondary outcomes are the implantation rate, miscarriage rate, and ectopic pregnancy rate. Implantation is confirmed by the ultrasound visualization of the embryo sac in the uterus. Ectopic pregnancy is defined when ultrasonographic visualization of gestational sacs is outside the uterine cavity. We confirmed the clinical pregnancy by ultrasonographic visualization of gestational sacs 28–35 days after embryo transfer. Miscarriage is defined as spontaneous clinical pregnancy loss before 28 gestational weeks.

### Statistical Analysis

The categorical variables were presented as percentages and frequencies; the continuous variables were presented as means ± standard deviations (SDs) or interquartile ranges (IQRs), depending on data distribution. We compared categorical variables between groups using the χ^2^ test, and we compared continuous variables using analysis of variance (ANOVA) or the Mann–Whitney test according to data distribution. We performed propensity score matching (PSM) to control confounding factors that may affect pregnancy outcomes. We perform PSM between the FC and NC groups at 1:1. The NC or FC group was the dependent variable. Independent variables included in the PSM model were maternal age, BMI, number of transferred embryos, number of transferred good-quality embryos, and embryo type. PSM was performed by the ‘MatchIt’ R package (29). The caliper was set as 0.02, and the method was set as “nearest” in the PSM. We set the random seed number as 12345678 to ascertain the statistical reproducibility. Three multivariate logistic models were performed to evaluate the effect of functional ovarian cyst on CPR by generalized estimation equation (GEE) with the ‘geepack’ R package (30). The GEE model was used because some patients performed more than one FET cycle. Variables included in model 1 were the number of transferred embryos, the number of transferred good-quality embryos, and the type of transferred embryos. Variables included in model 2 were the number of transferred embryos, the number of transferred good-quality embryos, the type of transferred embryos, maternal age, and BMI; Variables included in model 3 were the number of transferred embryos, the number of transferred good-quality embryos, the type of transferred embryos, maternal age, BMI, endometrial thickness, and endometrial pattern. In the multivariate logistic analysis, maternal age was divided into the following three groups: <30 years, ≥30 and <35 years, and ≥35 years. BMI was divided into the following four groups: <18.5 kg/m^2^, ≥18.5 and <22 kg/m^2^, ≥22 and <25 kg/m^2^, and ≥25 kg/m^2^. Endometrial thickness was divided into two groups: 7–10 mm and ≥10 mm. All statistical analyses were 2-sided, and we considered a *p*-value <0.05 to be statistically significant. All statistical analyses were performed by R (version 4.1.1, www.R-project.org).

## Results

### The Incidence Rate of GnRHa-Induced FC

A total of 599 patients who planned 657 HRT-FET cycles were included in the crude cohort. Sixty-four cycles were divided into the functional ovarian cyst group (FC), and 593 cycles were divided into the no functional ovarian cyst group (NC). The GnRHa-induced FC rate was 9.74% (64/657). A comparison between the NC and FC groups showed that female age was significantly higher in the FC group than in the NC group (33.56 ± 4.64 vs 31.40 ± 5.27; p-value = 0.001). BMI was significantly lower in the FC group compared to the NC group (20.93 ± 3.03 vs 22.24 ± 3.06; p-value = 0.002). The cancellation rate was significantly higher in the FC group compared to the NC group (7.81% vs 2.36%, p-value = 0.013). The infertility causes, basal FSH, endometrial thickness, and endometrial pattern were not significantly different between the FC and NC groups (p-values >0.05). The characteristics in patients of the crude cohort are shown in **Table 1**.

**Table 1:** Patients’ characteristics of the crude cohort.

	ALL (N = 657)	NC (N = 593)	FC (N = 64)	p-value
Age (year)	31.61 (5.25)	31.40 (5.27)	33.56 (4.64)	0.001
Infertility cause:				0.059
combined factor	164 (24.96%)	137 (23.10%)	27 (42.19%)	
DOR	28 (4.26%)	27 (4.55%)	1 (1.56%)	
male factor	85 (12.94%)	78 (13.15%)	7 (10.94%)	
ovulation factor	58 (8.83%)	53 (8.94%)	5 (7.81%)	
tubal factor	301 (45.81%)	278 (46.88%)	23 (35.94%)	
unknown factor	21 (3.20%)	20 (3.37%)	1 (1.56%)	
Endometrial thickness (mm)	9.56 (1.69)	9.55 (1.68)	9.64 (1.82)	0.722
BMI (kg/m^2^)	22.1 (3.08)	22.2 (3.06)	20.9 (3.03)	0.002
Basal FSH (mIU/ml)	6.29 [5.40;7.47]	6.25 [5.40;7.45]	6.73 [5.42;7.68]	0.200
Endometrial pattern:				0.234
A	151 (22.98%)	134 (22.60%)	17 (26.56%)	
B	461 (70.17%)	421 (70.99%)	40 (62.50%)	
C	45 (6.85%)	38 (6.41%)	7 (10.9%)	
ET Cancel rate	19 (2.89%)	14 (2.36%)	5 (7.81%)	0.013

FC, patients with functional ovarian cysts; NC, patients without functional ovarian cysts; DOR, diminished ovarian reserve; BMI, body mass index; FSH, Follicle-Stimulating Hormone; ET, embryo transfer.

### Risk Factors Associated With the Incidence of GnRHa-Induced FC

Patients with older age (aOR 1.10, 95% CI 1.05–1.15, p-value <0.001) and lower BMI (aOR 0.81, 95% CI 0.71–0.93, p-value = 0.002) are more likely to develop GnRHa-induced FC. Patients ≥35 had a significantly higher incidence rate of FC compared to patients <30 (aOR 3.21, 95% CI 1.61–6.39, p-value <0.001); 30≤ patients <35 had a higher incidence rate of FC compared to patients <30 without a significant statistical difference (aOR 1.70, 95% CI 0.86–3.35, p-value = 0.12). Patients with BMI <18.5 had a significantly higher incidence rate of FC compared to the patients with 18.5 ≤BMI <22 (aOR 2.52, 95% CI 1.22–5.22, p-value = 0.013); Patients with BMI ≥25 had a significantly lower incidence rate of FC compared to the patients with 18.5 ≤BMI <22 (aOR 0.32, 95% CI 0.11–0.95, p-value = 0.04); Patients with 22≤ BMI <25 had a similar incidence rate of FC compared to the patients with 18.5≤ BMI <22 (aOR 0.79, 95% CI 0.42–1.47, p-value = 0.50) (**Table 2**).

**Table 2:** Multivariate regression models analyzed the factors associated with the incidence of functional ovarian cysts.

		FC (N = 64)	NC (N = 593)	aOR	95% CI	p-value
Model 1§	Age	33.6 ± 4.64	31.4 ± 5.27	1.10	1.05–1.15	<0.001
	BMI	20.9 ± 3.03	22.2 ± 3.06	0.81	0.71–0.93	0.002
Model 2†	Age					
	Age <30	16 (25.0%)	226 (38.1%)	ref		
	30≤ Age <35	22 (34.4%)	220 (37.1%)	1.70	0.86–3.35	0.12
	Age ≥35	26 (40.6%)	147 (24.8%)	3.21	1.61–6.39	<0.001
	BMI					
	18.5≤ BMI <22	28 (43.8%)	242 (40.8%)	ref		
	22≤ BMI <25	19 (29.7%)	196 (33.1%)	0.79	0.42–1.47	0.50
	BMI <18.5	13 (20.3)	56 (9.44%)	2.52	1.22–5.22	0.013
	BMI ≥25	4 (6.25%)	99 (16.7)	0.32	0.11–0.95	0.04

FC, patients with functional ovarian cysts; NC, patients without functional ovarian cysts; aOR, adjusted odds ratio; CI, confidence interval; ref, reference; ^§^Variables as Age and BMI included in model 1 were continuous variables; ^†^Variables as Age and BMI included in models 2 were transformed into category variables.

### Baseline Characteristics and Pregnancy Outcomes Between the NC and FC Groups Before PSM

After exclusion, 544 cycles were included in the final analysis. Fifty-two cycles were divided into the FC group, and 492 cycles were divided into the NC group. A comparison of baseline characteristics between the two groups showed that female age was significantly higher in the FC group compared to the NC group (33.75 ± 4.72 vs 31.26 ± 5.17; p-value = 0.001) and BMI was significantly lower in the FC group compared to the NC group (21.04 ± 3.07 vs 22.04 ± 3.04; p-value = 0.029). The basal FSH and endometrial thickness were not significantly different between the FC and NC groups (p-values >0.05). A significantly higher proportion of patients in the FC group were transferred with good-quality embryos than those in the NC group (p-value = 0.009), while a significantly lower proportion of patients in the FC group were transferred with blastocysts than in the NC group (p-value = 0.037). Comparison of pregnancy outcomes between the two groups showed that the implantation rate (IR, 38.54% vs 38.95%, p-value = 0.958), clinical pregnancy rate (CPR, 55.77% vs 53.05%, p-value = 0.820), ectopic pregnancy rate (EPR, 1.92% vs 1.42%, p-value = 0.555), and miscarriage rate (MR, 5.77% vs 7.93%, p-value = 0.786) per frozen-thawed embryo-transferred cycle were not significantly different between the FC group and NC group. The baseline characteristics and pregnancy outcomes between the FC and NC groups before PSM are shown in **Table 3** (left four columns).

**Table 3:** Baseline characteristics and pregnancy outcomes between the FC group and the NC group before or after PSM.

	Before PSM			After PSM		
	NC (N = 492)	FC (N = 52)	p-value	NC (N = 42)	FC (N = 42)	p-value
Age	31.26 (5.17)	33.75 (4.72)	0.001	32.40 (5.17)	32.64 (3.99)	0.814
Infertility cause:			0.014			0.196
combined factor	85 (17.28%)	20 (38.46%)		9 (21.43%)	16 (38.10%)	
DOR	26 (5.28%)	0 (0.00%)		3 (7.14%)	0 (0.00%)	
male factor	71 (14.43%)	7 (13.46%)		6 (14.29%)	4 (9.52%)	
ovulation factor	51 (10.37%)	5 (9.62%)		3 (7.14%)	5 (11.90%)	
tubal factor	252 (51.22%)	20 (38.46%)		21 (50.00%)	17 (40.48%)	
unknown factor	7 (1.42%)	0 (0.00%)		0 (0.00%)	0 (0.00%)	
Endometrial thickness	9.72 (1.69)	9.79 (1.69)	0.760	9.51 (1.80)	9.96 (1.58)	0.222
Endometrial pattern:			0.030			0.114
A	118 (23.98%)	16 (30.77%)		8 (19.05%)	13 (30.95%)	
B	347 (70.53%)	29 (55.77%)		32 (76.19%)	23 (54.76%)	
C	27 (5.49%)	7 (13.5%)		2 (4.76%)	6 (14.29%)	
BMI	22.04 (3.04)	21.04 (3.07)	0.029	21.1 (2.57)	21.0 (2.43)	0.826
Basal FSH	6.30 [5.46;7.44]	6.67 [5.42;7.68]	0.315	6.29 [5.54;7.47]	6.86 [5.47;7.53]	0.434
Embryo type:			0.037			1.000
cleavage	301 (61.18%)	40 (76.92%)		32 (76.19%)	31 (73.81%)	
blastocyst	191 (38.82%)	12 (23.08%)		10 (23.81%)	11 (26.19%)	
No. transferred embryos:			0.161			0.770
1	124 (25.20%)	8 (15.38%)		6 (14.29%)	8 (19.05%)	
2	368 (74.80%)	44 (84.62%)		36 (85.71%)	34 (80.95%)	
No. Good Embryos:			0.009			0.529
0	68 (13.82%)	2 (3.85%)		2 (4.76%)	1 (2.38%)	
1	141 (28.66%)	9 (17.31%)		5 (11.90%)	9 (21.43%)	
2	283 (57.52%)	41 (78.85%)		35 (83.33%)	32 (76.19%)	
Implantation rate	335/860 (38.95%)	37/96 (38.54%)	0.958	24/78 (30.77)	28/76 (36.84%)	0.575
Clinical pregnancy rate	261 (53.05%)	29 (55.77%)	0.820	18 (42.86%)	23 (54.76%)	0.383
Miscarriage rate	39 (7.93%)	3 (5.77%)	0.786	3 (7.14%)	3 (7.14%)	1.000
Ectopic pregnancy rate	7 (1.42%)	1 (1.92%)	0.555	0 (0.00%)	1 (2.38%)	1.000

PSM, propensity score matching; FC, patients with functional ovarian cysts; NC, patients without functional ovarian cysts; DOR, diminished ovarian reserve; BMI, body mass index; FSH, Follicle-Stimulating Hormone; No., the number of.

### Baseline Characteristics and Pregnancy Outcomes Between the NC and FC Groups After PSM

After PSM, 42 cycles in the FC group were matched with 42 cycles in the NC group. A comparison of baseline characteristics showed that female age, BMI, infertility cause, basal FSH, the number of transferred embryos, the number of transferred good-quality embryos, and the type of transferred embryo were not significantly different between the two groups (p-values >0.05). Comparison of pregnancy outcomes showed that implantation rate (IR, 36.84% vs 30.77%, p-value = 0.575), clinical pregnancy rate (CPR, 54.76% vs 42.86%, p-value = 0.383), ectopic pregnancy rate (EPR, 2.38% vs 0.00%, p-value = 1.00), and miscarriage rate (MR, 7.14% vs 7.14%, p-value = 1.00) per frozen-thawed embryo-transferred cycle were not significantly different between the FC group and the NC group. The baseline characteristics and pregnancy outcomes between the FC and NC groups after PSM are shown in **Table 3** (right three columns).

### The Effect of HCG-Triggering GnRHa-Induced FC on Clinical Pregnancy in FET Cycles

Multi-regression Model 1, Model 2, and Model 3 showed that CPR is not significantly different between the FC group and the NC group when adjusting with different confounding factors (Model 1: aOR 1.18, 95% CI 0.64–2.17, p-value = 0.60; Model 2: aOR 1.47, 95% CI 0.75–2.86, p-value = 0.30; Model 3: aOR 1.56, 95% CI 0.79–3.08, p-value = 0.20) (**Table 4**).

**Table 4:** Association of HCG triggering GnRHa-induced FC and clinical pregnancy analyzed by three multivariate models adjusted with different confounders.

		Clinical pregnancy rate	aOR	95% CI	p-value
Model 1§	NC group	53.0%	ref		
	FC group	55.8%	1.18	0.64–2.17	0.60
Model 2†	NC group	53.0%	ref		
	FC group	55.8%	1.47	0.75–2.86	0.30
Model 3‡	NC group	53.0%	ref		
	FC group	55.8%	1.56	0.79–3.08	0.20

aOR, adjusted odds ratio; CI, confidence interval; ref, reference; §Adjusted for the number of transferred embryos, the number of transferred good-quality embryos, and the type of transferred embryos. †Adjusted for the number of transferred embryos, the number of transferred good-quality embryos, the type of transferred embryos, female age, and BMI. ‡Adjusted for the number of transferred embryos, the number of transferred good-quality embryos, the type of transferred embryos, female age, BMI, endometrial thickness, and endometrial pattern.

### Interactive Analysis and Subgroup Analyses

The interactive analysis showed that the effect of the HCG-triggering GnRHa-induced FC on clinical pregnancy is interactive with female age (p for interaction: 0.003). In subgroup analysis stratified by female age, multivariate regression analysis showed that HCG-triggering GnRHa-induced FC is significantly associated with a higher pregnancy rate than the NC group in patients ≥35 years (aOR 4.40, 95% CI 1.57–12.3, p-value = 0.005). However, in patients <35 years, the clinical pregnancy rate between the FC and NC groups was not significantly different (aOR 0.67, 95% CI 0.31–1.47, p-value = 0.30) (**Table 5**). Subgroup analysis based on embryo type showed that hCG triggering GnRHa-induced FC does not significantly affect the clinical pregnancy rate compared with the NC group in patients who transferred with D3 cleavage-stage embryos (aOR 1.84, 95% CI 0.95–3.58, p-value = 0.071) (**Table 5**). However, the sample size in the FC group transferred with D5 embryos is only 12. Considering the small sample size and insufficient statistical power, the subgroup analysis in the patients transferred with D5 blastocysts is not performed.

**Table 5:** Multivariate analysis showing the association between triggering GnRHa-induced FC with clinical pregnancy in subgroup of age ≥35, age <35, and embryo transferred = cleavages.

Subgroup	Treatment	n	aOR	95% CI	p-value
Age ≥ 35	NC group	118	ref		
	FC group	22	4.40	1.57–12.3	0.005
Age < 35	NC group	374	ref		
	FC group	30	0.67	0.31–1.47	0.30
Embryo = cleavages	NC group	301	ref		
	FC group	40	1.84	0.95–3.58	0.071

aOR, adjusted odds ratio; CI, confidence interval; ref, reference. The multivariate regression model was adjusted for the number of transferred embryos, the number of transferred good-quality embryos, the type of transferred embryos, female age, BMI, endometrial thickness, and endometrial pattern.

## Discussion

This study showed that HCG triggering of GnRHa-induced FC does not decrease the implantation rate and clinical pregnancy rate compared to the NC group in GnRHa + HRT-FET cycles but tends to increase the clinical pregnancy rate, especially in patients aged ≥35 years. Previous studies have proven that GnRHa-induced FC may contain mature follicles, and successful pregnancy can be achieved by HCG administration to trigger the oocyte maturation in fresh cycles (26–28). However, the feasibility and efficiency of HCG triggering of GnRHa-induced FC on FET outcomes remains to be elucidated. As far as we are aware, this is the first study that screens GnRHa-induced FC containing mature oocytes and evaluates the efficiency of HCG triggering functional ovarian cysts in HRT-FET cycles with 3.75 mg GnRHa pretreatment at the early follicular phase. The previous definition for functional ovarian cysts was: cyst diameter of >15 mm and an E_2_ of >50 pg/ml, with the purpose of evaluating the effect of functional ovarian cysts on fresh IVF outcomes (16). However, to investigate the feasibility of endometrial preparation by HCG trigger of GnRHa-induced FC, we set more strict criteria for screening functional ovarian cysts containing mature oocytes in our study: mean cyst diameter of ≥17 mm and an E_2_ of ≥100 pg/ml. Our study showed that the incidence rate of FC was 9.74%. Likewise, a previous study reported a similar incidence rate of functional ovarian cysts to be 9.3% at Day 14 of GnRHa downregulation with the criteria of cyst diameter of >15 mm and an E_2_ level of >50 pg/ml (16). Consistent with the previous studies, our study found older age to be one of the risk factors for GnRHa-induced FC (16, 31). However, we first report that lower BMI is another risk factor for GnRHa-induced FC. Although HCG trigger of GnRHa-induced FC does not affect FET outcomes in the general population, there is a tendency that HCG trigger of GnRHa-induced FC increases implantation rate (36.84% vs 30.77%) and clinical pregnancy rate (54.76% vs 42.86%) after PSM, though the p-value reached no statistical difference, probably due to small sample size. Moreover, interactive and subgroup analyses showed that the HCG trigger is associated with significantly higher CPR in patients ≥35. However, as the sample size in the age ≥35 subgroup is small, the results should be explained cautiously; a further larger sample size is needed to confirm the result. Older patients may be more sensitive to the endocrine perturbation caused by exogenous estrogen supplementation, while the HCG trigger of GnRHa-induced FC avoids exogenous estrogen use, which may be closer to the natural pregnancy state with less endocrine perturbation. Therefore, the HCG trigger of GnRHa-induced FC is more beneficial to patients older than 35 years.

In addition to HCG triggering, puncturing of the cysts, prolonging the GnRHa downregulation, and delaying exogenous estrogen supplementation are common treatments for GnRHa-induced FC. However, estrogen levels may drop rapidly after aspiration of the follicles (12) as the endometrium rapidly thins within 48 h of puncture (32). Furthermore, punctuation is a traumatic procedure that may lead to infection, bleeding, and other injuries (33). Prolonging the GnRHa downregulation and delaying exogenous E_2_ supplementation may cause E_2_ perturbation, leading to decreased endometrial receptivity and abnormal uterine bleeding, while canceling the cycle may increase the economic burden and the time to pregnancy.

It is worth noting is that, unlike traditional HRT regimens, the occurrence of ovarian hyperstimulation syndrome (OHSS) is possible if multiple FCs are triggered by HCG (26). In our study, among the 64 cycles with FC, five cycles developed more than three FCs; a patient with five follicles had the highest E_2_ level of 2,706 pg/ml. One patient with three follicles had an E_2_ level of 2,469 pg/ml, while the E_2_ levels of the other patients were below 1,000 pg/ml. None of them showed any symptoms of OHSS. However, we should be cautious when multiple follicles develop with extremely high levels of E_2_. Whether to give an HCG trigger or not should be decided carefully according to the E_2_ levels. It may be more reasonable to postpone exogenous estrogen supplementation until follicular atrophy or cancel the cycle or perform the oocyte pick-up and all embryo cryopreserved strategy.

In summary, HCG can trigger the GnRHa-induced FC if the mean diameter of the cysts is ≥17 mm, has a serum E_2_ level of ≥100 pg/ml, and an endometrial thickness of ≥7 mm after 14 days of GnRHa pretreatment in FET cycles, followed by luteal phase support and embryo transfer. It is not necessary to perform cyst punctuation or cancel the cycle. HCG triggering GnRHa-induced FC does not decrease the clinical pregnancy rate compared with NC in GnRHa-pretreated FET cycles. However, the following limitations of the study should be underlined: (1) The most significant limitation of this study is its retrospective nature. (2) The sample size is relatively small; further well-designed prospective study with a larger sample size is needed. (3) Other treatments, such as cyst punctuation and delaying exogenous supplementation, were not compared with HCG triggering due to small sample sizes.

## Data Availability Statement

The original data and statistical R scripts were deposited in our Github repositories (https://github.com/minizenghong/HCG_trigger_of_GnRHa-induced_FC).

## Ethics Statement

The study was performed in accordance with the Declaration of Helsinki. The study was approved by the Ethics Committee of Xiangya Hospital (accession number: 2021035).

## Author Contributions

HZ and CZ performed the statistical analysis, drafted the manuscript, tables, and figures; HZ, CZ, and LZ collected and cleaned the data, revised the tables and figures. NL designed the study and revised the manuscript. All authors listed have made a substantial, direct, and intellectual contribution to the work and approved it for publication.

## Funding

The study is supported by funds from the National Natural Science Foundation of China (https://www.nsfc.gov.cn/) (Grant number 81571441).

## Conflict of Interest

The authors declare that the research was conducted without any commercial or financial relationships that could be construed as a potential conflict of interest.

## Publisher’s Note

All claims expressed in this article are solely those of the authors and do not necessarily represent those of their affiliated organizations, or those of the publisher, the editors and the reviewers. Any product that may be evaluated in this article, or claim that may be made by its manufacturer, is not guaranteed or endorsed by the publisher.
